# Clonal Evolution at First Sight: A Combined Visualization of Diverse Diagnostic Methods Improves Understanding of Leukemic Progression

**DOI:** 10.3389/fonc.2022.888114

**Published:** 2022-07-08

**Authors:** Sarah Sandmann, Yvonne Lisa Behrens, Claudia Davenport, Felicitas Thol, Michael Heuser, Daniela Dörfel, Friederike Löhr, Agnes Castrup, Doris Steinemann, Julian Varghese, Brigitte Schlegelberger, Martin Dugas, Gudrun Göhring

**Affiliations:** ^1^ Institute of Medical Informatics, University of Münster, Münster, Germany; ^2^ Department of Human Genetics, Hannover Medical School, Hannover, Germany; ^3^ Department of Hematology, Hemostasis, Oncology and Stem Cell Transplantation, Hannover Medical School, Hannover, Germany; ^4^ Department of Hematology, Oncology and Immunology, Klinikum Region Hannover (KRH) Klinikum Siloah, Hannover, Germany; ^5^ Department of Hematology and Oncology, Klinikum Braunschweig, Braunschweig, Germany; ^6^ Hämato-Onkologische Praxis, Hämato-Onkologische Praxis im Medicum, Bremen, Germany; ^7^ Institute of Medical Informatics, Heidelberg University Hospital, Heidelberg, Germany

**Keywords:** clonal evolution, bioinformatics, single nucleotide variants, copy number variants, cancer cell fraction, leukemic progression

## Abstract

Patients with myeloid neoplasia are classified by the WHO classification systems. Besides clinical and hematological criteria, cytogenetic and molecular genetic alterations highly impact treatment stratification. In routine diagnostics, a combination of methods is used to decipher different types of genetic variants. Eight patients were comprehensively analyzed using karyotyping, fluorescence *in situ* hybridization, array-CGH and a custom NGS panel. Clonal evolution was reconstructed manually, integrating all mutational information on single nucleotide variants (SNVs), insertions and deletions (indels), structural variants and copy number variants (CNVs). To allow a correct integration, we differentiate between three scenarios: 1) CNV occurring prior to the SNV/indel, but in the same cells. 2) SNV/indel occurring prior to the CNV, but in the same cells. 3) SNV/indel and CNV existing in parallel, independent of each other. Applying this bioinformatics approach, we reconstructed clonal evolution for all patients. This generalizable approach offers the possibility to integrate various data to analyze identification of driver and passenger mutations as well as possible targets for personalized medicine approaches. Furthermore, this model can be used to identify markers to assess the minimal residual disease.

## Introduction

Myeloid neoplasia, including acute myeloid leukemia (AML) and myelodysplastic syndrome (MDS) are heterogeneous hematopoietic stem cell disorders, which are marked by the acquisition of somatic alterations and clonal evolution (1–4). Besides clinical and hematological criteria, cytogenetic and molecular genetic alterations highly impact treatment stratification (2). In recent years, high-throughput sequencing technologies have led to the identification of many driver and passenger alterations that enable precision medicine (e.g. *IDH1* inhibitor ivosidenib in AML patients) (2, 5–9). The different subgroups of myeloid neoplasia (e.g. MDS and AML) show a highly heterogeneous cytogenetic and molecular genetic profile. Multiple subclones may exist in one patient. Distribution of recurrently mutated genes and clonal architecture are different, e.g. in MDS/MPN subtypes (4, 10, 11). Consequently, leukemic progression may be influenced by several leukemic clones, and the clonal composition identified at diagnosis can differ from the composition identified at relapse or progression (12). Patients with myeloid neoplasia, especially patients with MDS, often progress to AML (sAML) through a process of clonal evolution (2, 11–13). Clonal evolution can be associated with poor prognosis, relapse and therapeutic resistance (14, 15). To this day different models of tumor evolution have been reported (e.g. linear, branching or neutral evolution) (16).

Recent publications have shown that reconstruction of clonal evolution and characterization of clonal architecture are important for the understanding of tumor development and treatment failure (14). Furthermore, they can help in finding new treatment strategies (e.g. combination of drugs, or new drugs for patients with relapse) (11, 12, 14). Different bioinformatic approaches already exist for the calculation of clonal evolution of bulk sequencing data. In addition, the single cell sequencing method has expanded the field of reconstructing clonal evolution (14, 17–19). A study of Morita et al. has shown both genetic and phenotypic evolution in AML by single cell sequencing and cell surface protein analyses (14). However, in routine diagnostics, a combination of methods (e.g. karyotyping and NGS panel) is used to identify alterations that are important for treatment stratification of patients with myeloid neoplasia, and which may not be detected using only a single method (20). These methods decipher different types of genetic variants, including single nucleotide variants (SNVs), insertions/deletions (indels), structural variants (SVs) and copy number variations (CNVs). The combination of a wide spectrum of methods could lead to the identification of many alterations, which, in turn, can make interpretation difficult. Unfortunately, an approach that combines all identified molecular genetic and cytogenetic aberrations and that reconstructs and visualizes the genetic architecture and clonal evolution, is still lacking.

In the present study, we performed karyotyping, fluorescence *in situ* hybridization, array-CGH and used a custom NGS panel in a cohort of eight patients with myeloid neoplasia. Here, we propose a bioinformatic approach as well as a visualization of occurrences of genetic alterations to improve our understanding of leukemic progression (driver and passenger alterations) and assist in finding the best treatment stratification paving the way for personalized medicine approaches. Clonal evolution and clonal architecture not only play a role in therapeutic resistance, relapse and poor outcomes in myeloid neoplasia, but also in other non-solid tumors (14). Our proposed bioinformatic model is a general approach, which may be used on all kinds on non-solid tumors showing clonal evolution.

## Materials and Methods

### Study Population

A total of eight patients with myeloid neoplasia and clonal evolution were included in this study. The patients were analyzed between 2011 and 2021 at our department. The ethical review boards of Hannover Medical School approved this study and all patients gave their written consent. Our cohort was analyzed at one or more time points. All results of performed analyses as well as clinical data are shown in **Table 1** and in more detail in **Table S1**.

**Table 1:** Overview of analyzed patients (n=8) (All results of performed analyses as well as clinical data are shown in **Table S1**).

patient (#)	WHO diagnosis	status	cytogenetic aberrations	DNA analysis NGS (somatic)			treatment
				gene	variant	variant allele frequeny (VAF)	
#1	AML^*1^	initial	del(5)(q14q33), -17, ​​add(18)(q22),​​+mar1,​​+mar2,​​+mar3	*ASXL1*	c.2317G>T p.(Glu773*)	10.83%	not known
				*DNMT3A*	c.2645G>A p.(Arg882His)	18.69%
				*IDH1*	c.394C>T p.(Arg132Cys)	13.85%
				*TP53*	c.427G>A p.(Val143Met)	20.49%
#2	MDS^*2^	initial	+8,​+10	*BCOR*	c.4639+1G>A	66.76%	Azacitidine plus Venetoclax
				*DNMT3A*	c.2645G>A p.(Arg882His)	39.43%
				*KRAS*	c.34G>C p.(Gly12Arg)	4.03%
				*SF3B1*	c.2098A>G p.(Lys700Glu)	1.31%
				*STAG2*	c.3362_3365dup p.(Ser1123Hisfs*14)	52.12%
				*U2AF1*	c.101C>T p.(Ser34Phe)	31.50%
#3	t-MN^*6^	initial	​t(9;20)(q11;q11),​t(12;22)(p13;q11)	*NF1*	c.2033del p.(Pro678Arg*10)	49.52%	CPY-351, allogenic stem cell planned
#4	t-MN	initial	del(5)(q14q34),+8,​i(8)(q10)x2	*IDH2*	c.419G>A p.(Arg140Gln)	46.79%	Azacitidine plus Venetoclax
				*RUNX1*	c.420T>G p.(Ser140Arg)	43.00%
				*SRSF2*	c.284C>T p.(Pro95Leu)	39.78%
				*TET2*	c.1455del p.(Asn486Thr*11)	47.20%
				*TET2*	c.3473del p.(Ala1158Glu*68)	41.43%
				*TP53*	c.844C>T p.(Arg282Trp)	90.92%
		progression	t(2;3)(p23;q27),​del(5)(q14q34),del(7)(q21),​+8,​i(8)(q10)x2	*IDH2*	c.419G>A p.(Arg140Gln)	33.28%
				*KRAS*	c.35G>C p.(Gly12Ala)	1.96%
				*NRAS*	c.35G>C p.(Gly12Ala)	1.70%
				*PTPN11*	c.1508G>C p.(Gly503Ala)	2.80%
				*RUNX1*	c.420T>G p.(Ser140Arg)	38.29%
				*SRSF2*	c.284C>T p.(Pro95Leu)	40.88%
				*TET2*	c.1398_1402dup p.(His468Leu*20)	5.11%
				*TET2*	c.1455del p.(Asn486Thr*11)	44.10%
				*TET2*	c.3473del p.(Ala1158Glu*68)	36.71%
				*TP53*	c.844C>T p.(Arg282Trp)	74.41%
#5	AML	initial	​​t(4;14;11)(q22;q32;q23),​​add(10)(p14)	*ASXL1*	c.2077C>T p.(Arg693*)	0.68%	7+3
				*KRAS*	c.38G>A p.(Gly13Asp)	0.35%
				*NRAS*	c.182A>G p.(Gln61Arg)	0.50%
		progression	​​t(4;14;11)(q22;q32;q23),​​+der(4)t(4;14;11),​+8,​+9,​add(10)(p14),​+19,​+21	*ASXL1*	c.2077C>T p.(Arg693*)	7.84%
				*KRAS*	c.38G>A p.(Gly13Asp)	7.59%
				*NRAS*	c.182A>G p.(Gln61Arg)	5.34%
#6	initial: MPN^*3^; 6-years later: suspicion on sAML^*4^	initial	–	*JAK2*	c.1849G>T p.(Val617Phe)	73.76%	Hydroxyurea & phlebotomy/blood letting; ~6 years later: Jakavi
		Progression	​add(5)(q12),​-7,​-13,​del(14)(q12q31),​der(17)t(13;17)(q21;p12)	*JAK2*	c.1849G>T p.(Val617Phe)	97.53%
				*TP53*	c.814G>A p.(Val272Met)	19.56%
		progression	​add(2)(q37),add(5)(q12),​-7,​-13,​del(14)(q12q31),​der(17)t(13;17)(q21;p12),​del(20)(q12q13)	*JAK2*	c.1849G>T p.(Val617Phe)	98.12%	11 cycles Azacytidin + 2 cycles LD Ara-C
				*TP53*	c.814G>A p.(Val272Met)	23.36%
#7	sAML from atypical CML^*5^	initial	​ins(9;12)(q34;p12p13),​+12	no variants detected			7+3 plus Dasatinib, allogenic Tx
		progression	+X,ins(9;12)(q34; p12p13), +11,​​+12,​​del(12)(p13),​+19	no variants detected		
		remission	-	no variants detected		
		relapse	der(7)t(7;9)(q35;q21),​​​ins(9;12)(q34;p12p13),​+12	no variants detected		
#8	AML without matu-ration	initial	​​del(9)(q21q31)	*NRAS*	c.37G>C p.(Gly13Arg)	12.69%	7+3, allogenic transplantation, after rezidive: FLA-V-IDA (=FLAG-IDA with Venetoclax) plus donor lymphocytes
				*WT1*	c.1136_1142dup p.(Ala382Thr*5)	23.26%
		remission	–	no variants detected		
		relapse	​​​​t(1;16)(p12;q21),del(9)(q21q31)	*NRAS*	c.37G>C p.(Gly13Arg)	12.78%
				*NRAS*	c.37G>T p.(Gly13Cys)	5.03%
				*WT1*	c.1141_1144dup p.(Ala382Val*4)	4.51%
				*WT1*	c.1136_1142dup p.(Ala382Thr*5)	11.35%
				*WT1*	c.1128dup p.(Thr377Asp*8)	1.48%
				*WT1*	c.1110dup p.(Val371Cys*14)	3.69%
		progression	t(1;16)(p12;q21),​​?add(9)(p12),​​del(9)(q21q31),​​add(10)(p12),add(17)(q22)	*NRAS*	c.37G>C p.(Gly13Arg)	17.38%
				*NRAS*	c.37G>T p.(Gly13Cys)	4.93%
				*WT1*	c.1141_1144dup p.(Ala382Val*4)	4.03%
				*WT1*	c.1136_1142dup p.(Ala382Thr*5)	17.18%
				*WT1*	c.1128dup p.(Thr377Asp*8)	4.76%
				*WT1*	c.1110dup p.(Val371Cys*14)	4.96%
		remission	–	no variants detected		

*1 AML, acute myeloid leukemia; *2 MDS, myelodysplastic syndrome; *3 MPN, myeloproliferative neoplasm; *4 sAML, secondary AML; *5 CML, chronic myeloid leukemia

### Karyotyping and Fluorescence *In-Situ* Hybridization (FISH) Analysis

Cytogenetic and molecular cytogenetic analyses were performed on bone marrow aspirates or peripheral blood cultures. Chromosome preparation and fluorescence R-banding were performed as described previously (21, 22). Altogether, whenever possible, 15 to 25 metaphases were examined per patient. The karyotype was described according to guidelines of the International System for Human Cytogenetic Nomenclature (23). Depending on the cytogenetic aberrations in each patient, FISH analyses on interphase nuclei were performed using: (1) break apart probes for the loci 3q26 (EVI1 (MECOM) Break Apart; Cytocell, Cambridge, UK), 11q23 and 12p13 (Vysis MLL and ETV6 FISH Probe Kit; Abbott, IL, USA); (2) dual color probes for the loci 5p15.2, 5q31, CEP7, 7q31, 9q34.1, 17p13.1, CEP17, 22q11.2 (Vysis EGR1/D5S23, D5S721, D7S486/CEP7, BCR/ABL and TP53/CEP 17 FISH Probe Kit; Abbott); and (3) single color probe for the locus 20q12 (Vysis D20S108 FISH Probe Kit; Abbott). At least 200 interphase nuclei were analyzed for each probe.

### Array-CGH (Array-Based Comparative Genomic)

According to the manufacturer’s protocol (e-Array design 84704, Agilent Technologies, Waldbronn, Germany), three patients (patients c1, 6 and 7) were screened for CNVs by high-resolution array-CGH. Microarray slides were scanned immediately using an Agilent microarray scanner at a resolution of 2 μm. Fluorescence ratios were calculated using Feature Extraction Software and copy number states analyzed using the CGH data analysis software Genomic Workbench (Agilent Technologies).

### NGS With IDT Custom Panel

DNA sequencing was performed for all patients using an IDT custom panel (Integrated DNA Technologies Inc, Iowa, USA) including 148 leukemia-associated genes. Sequencing was performed on an Illumina NextSeq sequencer. All samples were aligned against the reference genome GRCh37 using BWA mem (24). The pipeline uses eight open-source tools for independent variant calling. Subsequently, raw calls are automatically combined and characterized with respect to data quality. Default settings were used (minimum number of reads 50, minimum number of reads with the alternate allele 20, minimum variant allele frequency VAF 0.01). Excluding low-quality calls, the remaining variants are further characterized (e.g. presence in common databases and in silico effect prediction). Combining all information, an artifact- and a polymorphism score is calculated for every SNV and small indel. The scores allow for automatic classification of the calls as true variants, polymorphisms and artifacts. Additionally, all detected variants were manually checked using the Integrative Genomics Viewer (IGV) to confirm automatic classification (25, 26). All detected variants were classified according to the standards and guidelines of the American College of Medical Genetics and Genomics (ACMG) (27). Here, we report only variants of unknown significance (VUS), likely pathogenic and pathogenic variants.

### Bioinformatic Approach

SNVs and short indels were detected using targeted NGS and variant calling pipeline appreci8 (28). SVs and CNVs were detected performing karyotyping, FISH and array-CGH analysis. Clonal evolution was reconstructed manually, integrating all mutational information on SNVs, indels, SVs and CNVs as described in Reutter et al. (29). The results were visualized by fishplots (30). In short, percentages of cancer cell fractions (CCFs) for SNVs and indels were estimated based on VAFs, assuming heterozygous variants (2*VAF=CCF). Percentages of CCFs for SVs and CNVs were estimated based on cell counts reported for karyotyping and FISH analyses. For SVs and CNVs that were only detected in array-CGH percentages of CCF were estimated based on log_2_Ratio of fluorescence intensities for reference versus tumor probes. In case of CNVs overlapping the position of an SNV or indel, the calculation of CCF is less straightforward. Altogether, we differentiate between three possible scenarios:

Scenario 1: The CNV occurred prior to the SNV/indel, but in the same cells.
Scenario 2: The SNV/indel occurred prior to the CNV, but in the same cells.
Scenario 3: SNV/indel and CNV exist in parallel, independent of each other.

**Figure 1:**
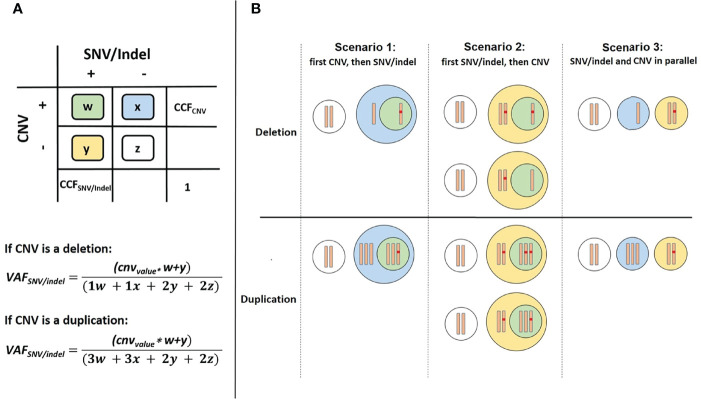
Overview of cell types in case of a CNV overlapping the position of an SNV/indel. **(A)** Color scheme used for 4 possible cases. **(B)** Differentiation between the three possible scenarios.

We defined *w* as the ratio of cells featuring a CNV and an SNV/indel. Analogously, *x*, *y* and *z* were defined in the same manner. The sum over all cell ratio was always *w+x+y+z=1.0*. Furthermore, the known CCF for the CNV was defined as *CCF_CNV_=w+x*. Additionally, two formulas were derived from the model (see **Figure 1A**):


(1) $$VAF_{SNV/indel}=\left(cnv_{value}\cdot w+y\right)/\left(1\cdot w+1\cdot x+2\cdot y+2\cdot z\right)\;if\;CNV\;is\;deletion\;$$

(2) $$VAF_{SNV/indel}=\left(cnv_{value}\cdot w+y\right)/\left(3\cdot w+3\cdot x+2\cdot y+2\cdot z\right)\;if\;CNV\;is\;duplication$$

We defined *cnv_value_* as a parameter to model the number of alleles with the SNV/indel depending on the CNV. For scenarios 1 and 3 the *cnv_value_=1* and for scenario 2 the *cnv_value_* can be 0, 1 or 2. Dependent on the three scenarios we considered, the two equations may be further simplified: For scenario 1, the CNV occurring prior to the SNV/indel, *y=0* (see **Figure 1B**). Thus, *CCF_CNV_=w+x* and *CCF_SNV/indel_=w*. As *VAF_SNV/indel_* is also known, *w* and *CCF_SNV/indel_* can be easily determined. For scenario 2, a distinction of cases has to be made. Both, the allele with or without SNV/indel may be affected by CNV. Regarding deletions, *cnv_value_=0* if the allele with SNV/indel is deleted, and *cnv_value_=1* if the allele without SNV/indel is deleted. Regarding duplications, *cnv_value_=2* if the allele with SNV/indel is duplicated, and *cnv_value_=1* if the allele without SNV/indel is duplicated. Additionally, *x=0*, so *CCF_CNV_=w*. The unknown value of y and thus *CCF_SNV/indel_=w+y* can be determined using *VAF_SNV/indel_*, following the distinction of cases. For the third scenario of SNV/indel and CNV occurring in parallel, independent of each other, *w=0*. Thus, *CCF_CNV_=x* and *CCF_SNV/indel_=y*. As *VAF_SNV/indel_* is known, the unknown value of *CCF_SNV/indel_* can be determined. Although *CCF_SNV/indel_* can always be determined using the above-mentioned formulas, the result may not always be distinct. Information on whether the CNV occurred prior or after the SNV/indel is in general not available. Therefore, for application of our approach, all scenarios have to be considered and four possible values for *CCF_SNV/indel_* have to be calculated. Scenarios may be excluded if the basic assumption *w+x+y+z=1.0* is violated.

## Results

Eight patients with myeloid neoplasia were comprehensively analyzed using karyotyping, fluorescence *in situ* hybridization (FISH), and a custom NGS panel. In addition, array-CGH analysis was performed whenever possible. All patients showed clonal evolution with many genomic variants, either at the initial time point and/or during the course of the disease.

### Identification of Linear Clonal Evolution in Patients Analyzed at One Time Point

**Figure 2:**
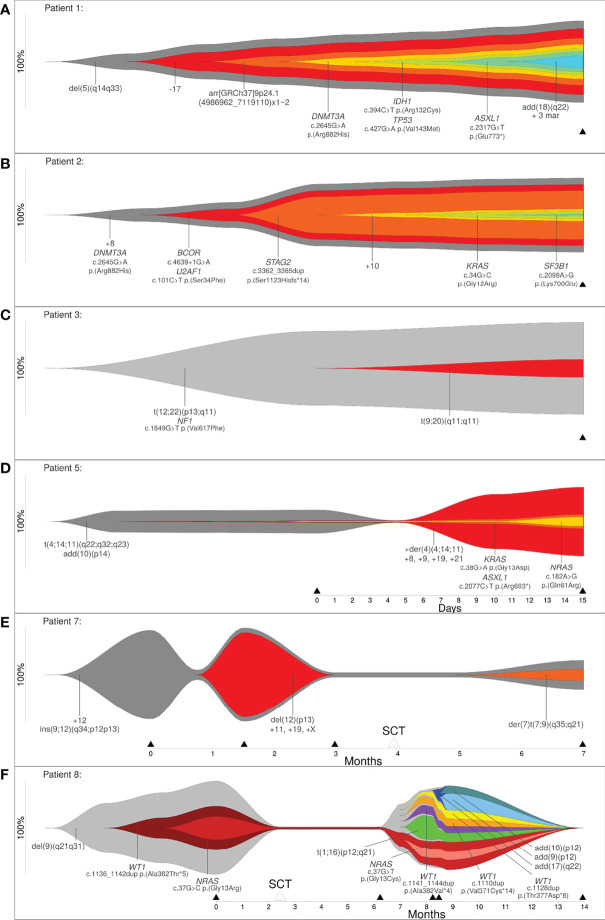
**(A–C)** Reconstruction of clonal evolution of three patients analyzed at one time point. **(D–F)** Clonal evolution patterns of patient 5 (linear evolution), patient 7 (branched evolution) and patient 8 (neutral evolution). Y-axis represents the percentage of clone size, black triangles indicate the analyzed time point and white triangles indicate time point of stem cell transplantation.

In order to show that clonal evolution can be reconstructed and visualized even when only one time point is available, we examined three patients (patient 1, 2 and 3) with the aforementioned bioinformatic approach. The total number of aberrations in a patient ranged from 3 (patient 3) to 11 (patient 1). Due to this approach it was possible to determine clones by clustering mutations. These clones were sorted according to CCF, in order of their appearance. Through reconstruction and visualization of clonal evolution it was possible to identify the initial clone (stemline) as well as the passenger aberrations for all patients. For example, patient 1 (AML) showed a deletion in 5q (del(5)(q14q33)) in the initial clone, patient 2 (MDS) showed a trisomy 8 as well as a pathogenic variant in *DNMT3A* in the initial clone, and patient 3 (therapy related myeloid neoplasm) showed a translocation t(12;22) in combination with a pathogenic variant in *NF1* in the initial clone (see **Figure 2** and **Table 1**/**S1**). The total number of clones in a patient ranged from 2 (patient 3) to 7 (patient 1). In summary, our approach was successful in identifying linear clonal evolution in all patients.

### Identification of Different Clonal Evolution Models (Linear, Branching or Neutral) in Patients Analyzed at More Than One Time Point

Next, we analyzed five patients where data was available for more than one time point (2 to 5 time points of analysis) to show that the bioinformatic approach can reconstruct different models of clonal evolution. At each time point, all patients were analyzed by a combination of methods, including karyotyping, FISH and a custom NGS-Panel. The estimated therapy effect was included in the reconstruction of clonal evolution. The approach reconstructed a distinct model of clonal evolution for patients 5, 7 and 8 (see **Figures 2C–E**). Patient 5 was analyzed at two time points – initial and progression. The bioinfomatic approach reconstructed a linear clonal evolution pattern. The initial clone showed a translocation t(4;14;11) and a structural aberration in chromosome arm 10p (add(10)(p14)). At this time point, pathogenic variants in *KRAS*, *ASXL1* and *NRAS* were identified with a very low percentage of CCF (close to 1%). However, the pathogenic variants were only identified by deep sequencing. At the second time point (progression), additional chromosome aberrations (+der(4)(4;14;11), +8, +9, +19 and +21) were identified and this subclone became the mainline (see **Figure 2D**). Patient 7 was analyzed at four time points – initial, progression, remission and relapse. Stem cell transplantation was performed between the third and fourth time point of analysis. The initial clone showed an insertion of a segment of chromosome 12 into chromosome 9 (ins(9;12)) and a trisomy 12. At time point two, the percentage of cells only harboring the stemline’s mutations were greatly reduced (~5%) and a subclone developed. This clone showed a deletion in 12p (del(12)(p13)) and a trisomy 11, 19 and X. For the subclone, the approach calculated linear clonal evolution. At time point three, the subclone was no longer detectable, but a new subclone was identified at time point four. This subclone presented a derivative chromosome 7 with a translocation t(7;9) (der(7)t(7;9)(q35;q21)). At this time point, the clonal evolution pattern of patient 7 changed from linear to branched (see **Figure 2E**). Patient 8 was analyzed at five time points – initial, remission, progression, relapse and remission. Stem cell transplantation took place between the first and second time point. The initial clone showed a deletion in 9q (del(9)(q21q31)) and a subclone with a likely pathogenic variant in *WT1* as well as a pathogenic variant in *NRAS*. At this time point, a linear evolution pattern was reconstructed. At time point two, after stem cell transplantation, no variants were detected – neither by karyotyping nor deep sequencing. However, the aforementioned variants were present at time point three (progression). At this time point, a linear clonal evolution pattern was reconstructed in one subclone (light red). Furthermore, four additional subclones were observed. All subclones developed independently of the initial clone with a deletion in 9q (del(9)(q21q31)). At the fourth time point of analysis, three additional subclones were detected. The clonal evolution pattern of patient 8 changed from linear to neutral. At the last time point of analysis (remission), no variants were detected (see **Figure 2F**). In summary, a linear evolution pattern was reconstructed for patient 5, a branched evolution pattern was reconstructed for patient 7 and a neutral evolution pattern was reconstructed for patient 8.

**Figure 3:**
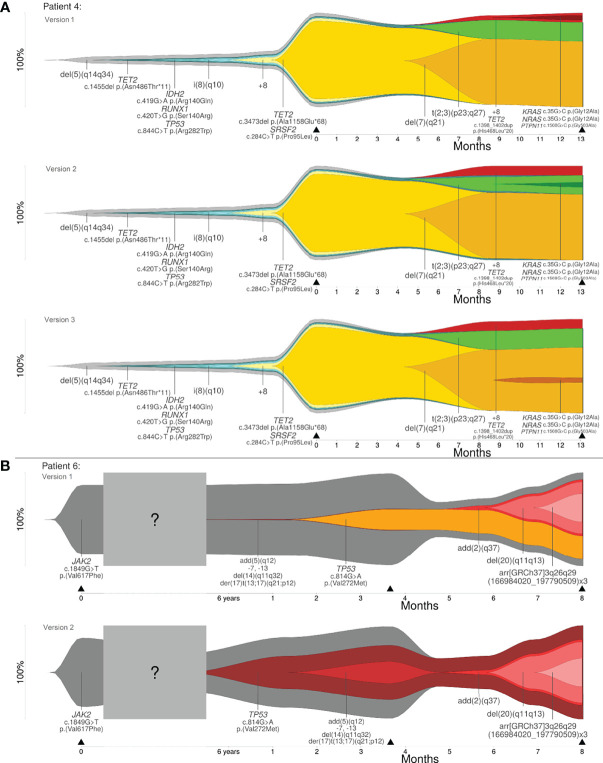
**(A)** Clonal evolution patterns of patient 4: three versions of branched evolution. **(B)** Clonal evolution patterns of patient 6: linear and branched evolution. Y-axis represents the percentage of clone size and black triangles indicate the analyzed time point.

Reconstruction of clonal evolution in patients 4 and 6 showed more than one possible clonal evolution pattern. All possible versions of reconstruction are shown in **Figure 3**. Patient 4 was analyzed at two time points (initial and progression). The initial clone showed a deletion in 5q (del(5)(q14q34)). Additionally, four subclones developed. At this time point, patient 4 showed a linear clonal evolution pattern. At time point two, four additional subclones were detected and the evolution pattern changed from a linear to a branched evolution pattern. Although the model of clonal evolution might be clear, it was not possible to reconstruct the precise subclonal relationship. The smallest subclone with (likely) pathogenic variants in *KRAS*, *NRAS* and *PTPN11* showed a low percentage of CCF (5%). Based on the available data, the parent clone of the smallest subclone cannot be definitely determined and, therefore, patient 4 showed three possible versions of branched evolution patterns (see **Figure 3A**). Patient 6 was analyzed at three time points – initial and two progression time points. The initial clone showed a pathogenic variant in *JAK2*. At the second time point of analysis, we identified two events: (1) a likely pathogenic variant in *TP53* and (2) a derivative chromosome 17 resulting in loss of *TP53* (der(17)t(13;17)(q21;p12)) with further aberrations. Based on the available data, it was not possible to reconstruct which of the two events took place first. Furthermore, it was not possible to calculate if both events affected the same allele or different alleles. As described in Materials and Methods, four different cases have to be considered (see **Figure 1A**). Two out of four cases were in line with the basic assumption w+x+y+z=1.0. Both cases were confirmed by the observed percentages of CCF and VAFs at the time points one and two of analysis. According to the first case, the CNV event took place first, followed by the likely pathogenic variant in *TP53*. Both aberrations affected different alleles. For this case, a branched clonal evolution pattern was reconstructed (see **Figure 3B**). Mathematically, it is also possible that the point mutation in *TP53* took place first and subsequently the mutated allele was affected by the deletion. For this case, a linear clonal evolution pattern was reconstructed. However, a biallelic inactivation of *TP53* frequently occurs during disease progression of hematologic neoplasms (e.g. MDS) (31). In summary, three possible branched evolution patterns were reconstructed for patient 4, and two models of clonal evolution (linear and branching) were reconstructed for patient 6.

## Discussion

In this study we propose a bioinformatic approach to analyze clonal evolution and the genetic architecture comprising SNVs, indels and SVs, especially CNVs in patients with myeloid neoplasia. In order to show the possibility of reconstruction and visualization of clonal evolution at one time point of analysis, we analyzed three patients with myeloid neoplasia. Here, all patients showed a linear clonal evolution pattern. Furthermore, with this bioinformatic approach, we addressed all alterations that may play a role in the pathogenesis of the disease (driver) and alterations, which may occur during disease development (passenger). For example, patient 1 and 2 showed a *DNMT3A* ^R882^ mutation at the initial time point of analysis. Pathogenic variants in *DNMT3A* occur in ~30% of AML patients and studies of clonal architecture have shown that pathogenic variants in *DNMT3A* occur as an early event in leukemogenesis. For that reason, *DNMT3A* could be a possible therapeutic target in the future (32).

In recent years, several targeted therapies have been approved for the treatment of patients with myeloid neoplasia. For example, the development of genome sequencing has identified numerous somatic alterations in patients with AML. Some of these alterations (“actionable mutations”) can be targeted by specific drugs to improve the outcome of the patients (33, 34). For example, patient 1 had an *IDH1* ^R132^ mutation, which can be found in 5-10% of AML patients. Patients with an *IDH1* ^R132^ mutation can be treated with ivosidenib. A recent study has shown that in these patients single-agent ivosidenib treatment leads to a complete remission (CR) or CR with incomplete haematological recovery rates of 42.4% (5). A phase trial study has analyzed a cohort of AML patients with a pathogenic variant in *IDH1*, which were treated with ivosidenib-and-azacitidine or with placebo-and-azacitidine. Patients treated with ivosidenib-and-azacitidine showed a significantly longer event-free survival and overall survival (24.0 month vs. 7.9 month) in comparison to patients treated with placebo-and-azacitidine (35). In addition, Bolton et al. have analyzed the clonal hematopoiesis in patients with therapy-related myeloid neoplasms. The study has shown that pathogenic variants show a different clonal evolution based on exposures (e.g. radiation, topoisomerase II inhibitors), for example, clones with pathogenic variants in DNA damage response genes outcompetes other clones (36). Thus, reconstruction of clonal evolution as well as identification of driver and passenger alterations allow a better understanding of the mechanisms of leukemogenesis and improvement of (future) treatment strategies (personalized therapy).

We analyzed five patients at more than one time point to show that the approach can reconstruct different models of clonal evolution (i.e. linear, branching and neutral evolution). For all of these patients the estimated therapy effect was included. The analyzed data of three out of these five patients allowed a distinct reconstruction of clonal evolution (patients 5, 7 and 8). Linear evolution is characterized by a dominant clone which overgrows the ancestral clone after acquisition of additional mutations in a stepwise manner (3, 4) - a linear mutation pattern was reconstructed for patient 5. Branching evolution is characterized by the occurrence of different subclones from on ancestral clone (3, 4) – as detected in patient 7. Neutral evolution is an extreme case of branching evolution, which is characterized by the accumulation of random mutations over time (16) – as was observed in patient 8. A possible advantage of reconstructing clonal evolution could be to show if both pathogenic variants co-occur in the same clone or in different clones. This could influence the treatment strategy. For example, patient 8 showed a pathogenic variant in *NRAS* and in *WT1*. Pathogenic variants in *NRAS* occur in ~12% of all AML cases and co-occur with pathogenic variants in epigenetic modifiers (*TET2/IDH/WT1*). The co-occurrence of pathogenic variants in *NRAS* and *WT1* showed a sensitivity to MAPK kinase inhibition, which has been shown in patient samples and mouse models providing a possible treatment strategy in the future (32). Especially patients with AML show a high number of heterogeneous pathogenic variants at diagnosis and relapse. These pathogenic variants are organized into a hierarchy of clones and they are able to adapt and evolve in response to therapeutic pressure (5). In the future, the clonal evolution pattern and genetic architecture of many patients must be analyzed in order to learn more about therapy resistance and new possibilities for therapy strategies to improve patient outcome.

For the remaining two patients (patient 4 and 6) the analyzed data showed more than one possible clonal evolution pattern. The proposed bioinformatic approach can be used to analyze the order and occurrence of genetic alterations, which could be relevant for treatment decisions and for the understanding of leukemic progression. For example, patient 6 showed a *JAK2/TP53* clone at the transformation to AML, which replaced the dominant *JAK2* clone in the myeloproliferative neoplasia (initial diagnosis). This observation is in concordance with a recent publication, where a significant alteration in clonal architecture or a “clonal sweep” with emergence of new dominant clone(s) was observed with single cell sequencing (17). These observations can be used for therapy stratification during the disease course.

Altogether, we present a generalizable approach that enables the combination of a wide spectrum of methods and to analyze retrospective samples to visualize leukemic progression and genetic architecture. Recent publications have reconstructed clonal evolution with single cell sequencing where the available data has allowed definitive identification of clonal architecture (17, 19, 37). Single cell sequencing has many advantages: exact distinction of different clones, measurement of accurate clonal complexity or resolution of mutational order (17, 38). But there are also many disadvantages, including limited depth of sequencing, no possibility of analyzing retrospective samples (no suitable material), or missing information of chromosomal aberrations (important for treatment stratification). Even though the used methods in this project do not offer the possibility to analyze clonal evolution at a single cell level, the inclusion of cytogenetic and molecular genetic alterations allow the identification of driver and passenger alterations, which is currently impossible using only single cell sequencing. For future projects, a combination of methods (bulk and single cell), including karyotyping and single cell sequencing, could be helpful for the exact reconstruction of clonal evolution.

In conclusion, this bioinformatic approach offers the possibility of analyzing clonal evolution and the order and occurrence of many cytogenetic and molecular genetic alterations (genetic architecture) at one or more time points of analysis. Different models of clonal evolution (i.e. linear, branching and neutral) can be reconstructed with this approach. As the approach describes integration of data for reconstruction of clonal evolution in general, its application is not limited to myeloid neoplasms. Instead, it may be applied on all kinds of non-solid tumors showing clonal evolution. The visualization of the results in fishplots contributes to a better understanding of genetic architecture and leukemic progression. This approach helps to identify possible targets for the disease (personalized therapy) and can be used to identify markers in order to assess minimal residual disease.

## Data Availability Statement

The original contributions presented in the study are publicly available. This data can be found here: PRJNA847150 link to https://www.ncbi.nlm.nih.gov/sra/PRJNA847150.

## Ethics Statement

The study was conducted according to the guidelines of the Declaration of Helsinki. The ethical review boards of Hannover Medical School approved this study (Nr. 8657_BO_K_2019) and all patients gave their written consent. Informed consent was obtained from all subjects involved in the study. Reported results can be found in **Supplementary Materials**.

## Author Contributions

The study was conceived by SS, YB, and GG. Data and material of the study was generated by YB, GG, SS, DS, FT, MH, AC, DD, FL, JV, MD, and BS. Data collection was performed by SS, YB, GG. Interpretation and analysis were conducted by YB, GG, SS, MD, and BS. SS and YB wrote the manuscript. The paper was edited by all authors. All authors contributed to the article and approved the submitted version.

## Funding

GG and BS have been supported by the BMBF MyPred (01GM1911A).

## Conflict of Interest

The authors declare that the research was conducted in the absence of any commercial or financial relationships that could be construed as a potential conflict of interest.

## Publisher’s Note

All claims expressed in this article are solely those of the authors and do not necessarily represent those of their affiliated organizations, or those of the publisher, the editors and the reviewers. Any product that may be evaluated in this article, or claim that may be made by its manufacturer, is not guaranteed or endorsed by the publisher.
